# Modelos fotoelásticos aplicados a la restauración dental

**DOI:** 10.21142/2523-2754-0904-2021-084

**Published:** 2021-12-09

**Authors:** Miriam Marín-Miranda, María Lilia Juárez-López, Adrián Espinosa-Bautista

**Affiliations:** 1 Facultad de Estudios Superiores Zaragoza, Universidad Nacional Autónoma de México. Ciudad de México, México. miriam.marin@zaragoza.unam.mx, liadju@yahoo.com Universidad Nacional Autónoma de México Facultad de Estudios Superiores Zaragoza Universidad Nacional Autónoma de México Ciudad de México Mexico miriam.marin@zaragoza.unam.mx liadju@yahoo.com; 2 Facultad de Ingeniería, Universidad Nacional Autónoma de México. Ciudad de México, México. adrianeb@unam.mx Universidad Nacional Autónoma de México Facultad de Ingeniería Universidad Nacional Autónoma de México Ciudad de México Mexico adrianeb@unam.mx

**Keywords:** educación en odontología, simulación, biomecánica, operatoria dental, dental education, simulation training, biomechanical phenomena, dentistry operative

## Abstract

**Objetivo:**

Mejorar la comprensión de los cambios generados por los procedimientos restaurativos a través de modelos fotoelásticos. La reconstrucción dental debe fundamentarse en principios de oclusión para no provocar puntos prematuros de contacto durante los procedimientos de preparación y restauración que pueden provocar trastornos como trauma oclusal, disfunción de la ATM o fracturas. En este documento se presenta un método didáctico para visualizar de forma objetiva las cargas oclusales y su distribución.

**Materiales y métodos:**

Se realizaron modelos fotoelásticos de dientes y segmentos maxilares, para visualizar los efectos de las preparaciones dentales y las restauraciones, así como el desgaste de los puntos prematuros de contacto.

**Resultados:**

Por fotoelasticidad, se analizó la dirección, intensidad y distribución de esfuerzos en la corona y el soporte óseo.

**Conclusiones:**

La técnica permite visualizar cómo se afectan las estructuras desde la preparación cavitaria, los diferentes esfuerzos con fresas de diamante o carburo, además de observar cómo un punto prematuro de contacto no solo afecta al órgano que lo contiene, sino las estructuras aledañas.

## Abstract (en)

**Objective:**

To improve the understanding of the changes generated by restorative procedures through photoelastic models. Dental reconstruction should be based on principles of occlusion in order not to cause premature contact points during preparation and restoration procedures that can lead to disorders such as occlusal trauma, temporo-mandibullary joint dysfunction, or fractures. This paper presents a didactic method to objectively visualize occlusal loads and their distribution.

**Materials and methods:**

Photoelastic models of teeth and maxillary segments were made to visualize the effects of dental preparations and restorations, as well as the wear of premature contact points.

**Results:**

The direction, intensity and distribution of stresses in the crown and bone support were analyzed by photoelasticity.

**Conclusions:**

The technique allows visualization of how the structures are affected from the cavity preparation, the different stresses with diamond or carbide burs, in addition to observing how a premature contact point not only affects the organ that contains it but also the surrounding structures.

## INTRODUCCIÓN

La preparación de cavidades y la restauración directa conforman el día de la práctica profesional de los cirujanos dentistas, por lo que conocimientos básicos sobre anatomía dental y fundamentos de oclusión son indispensables para lograr restaurar la forma y función del sistema estomatognático integralmente. 

Los huesos maxilares, dientes, articulación temporomandibular (ATM), músculos, ligamentos y sistema vasculo-nervioso constituyen una unidad funcional. Si alguno de estos elementos sufre modificaciones repercute en todo el sistema. Para los dientes, la forma, la relación de las caras oclusales y los bordes incisales deben ser precisos para lograr el equilibrio del sistema ^1^.

Al restaurar los tejidos dentarios, lo ideal es devolver forma y función. Modificaciones sutiles, en escala micrométrica y casi imperceptibles para el ojo humano pueden generar un desequilibrio que, al principio, genera dolor leve, pero si persiste, puede generar lesiones periodontales o articulares. Una estrategia para prevenir dichas patologías es comprender los mecanismos que las originan. 

La fuerza ejercida durante la masticación se transfiere desde los órganos dentarios, el ligamento periodontal y el soporte óseo hasta la ATM, de manera que es importante conocer y analizar estos esfuerzos. Para analizar el comportamiento físico de los cuerpos por medio de cálculos matemáticos y geométricos, se han usado ensayos mecánicos y método de elementos finitos, pero su uso requiere de equipo sofisticado y conocimientos especializados, además de presentar algunas discrepancias en el caso de modelos de formas complejas ^2^^,^^3^.

Una alternativa es la fotoelasticidad, ya que permite observar esfuerzos en modelos 3D complejos. Por su practicidad, se ha aplicado con éxito en la enseñanza de la ingeniería donde ya se ha validado su uso desde hace tiempo.

El cuerpo del hombre ha evolucionado en su forma de acuerdo con las necesidades y exigencias físicas que se le presentan. Así, se han modificado los huesos craneales por la ventilación y masticación, para ejecutar sus funciones de forma más eficiente. Las formas, tamaños y posiciones de cada cavidad neumática, cóndilo mandibular, cavidad glenoidea y cúspides presentes en los órganos dentarios se deben a la necesidad de cubrir una serie de movimientos de apertura y cierre, protrusión, retrusión y lateralidad; por supuesto la forma e inserción muscular también se ha visto modificada. 

Para estudiar las relaciones entre cada estructura del sistema desde el punto de vista físico, recurrimos a la biomecánica, apoyada por la cinética, la cinemática y la anatomía funcional para analizar el movimiento. Gracias a ella se pueden explicar los movimientos mandibulares, la participación muscular y las variaciones anatómicas en cada grupo dental ^1^. 

Por otro lado, las propiedades de los tejidos también son modificadas de acuerdo con su función. Los huesos y dientes son anisotrópicos, es decir, se modifican con respecto a la demanda mecánica, de tal forma que la orientación de los prismas del esmalte, los túbulos dentinales o trabeculado óseo, su grosor o densidad son diferentes de acuerdo con la exigencia física ^2^. Durante la masticación se aplican hasta 200 kg/cm^2^ en zonas de alto requerimiento. Las cargas se distribuyen y amortiguan desde los dientes en varios puntos hasta el cráneo. Primero en el esmalte dental hasta la dentina; ahí la distribución del esfuerzo depende la forma, la orientación y la elasticidad de cada tejido. El segundo punto son las fibras periodontales, insertadas en diferentes orientaciones hacia el hueso periodontal. El tercer punto amortiguador es el hueso esponjoso donde lo principal es la orientación del trabeculado. Los esfuerzos restantes son distribuidos en el maxilar hasta las cavidades neumáticas y la bóveda craneal, y en la mandíbula por toda la rama hasta la ATM. 

En los dientes, cada grupo tiene requisitos diferentes por su función, el mayor compromiso en la zona de corte se da en los centrales, en la de desgarre en caninos y premolar inferior y en la zona de trituración en los primeros molares. Esto además de tener relación con el grupo dental, también determina el número de raíces presentes ^3^.

Tratándose del sistema estomatognático, la palabra “oclusión” se refiere tanto a la relación cúspide-fosa, como a la alineación de las arcadas y los diferentes movimientos funcionales como parte de un todo. Así, para todo tratamiento restaurativo se deben tomar en cuenta aspectos como las cúspides de apoyo, las contenciones céntricas, los declives y la guía incisal ^4^. Los conceptos fundamentales de la oclusión a tomar en cuenta para la rehabilitación dentaria incluyen la relación céntrica: relación máxilo-mandibular donde los cóndilos están en una posición centrada, más superior y posterior que la cavidad glenoidea, y la oclusión céntrica: cuando la oclusión coincide con la relación céntrica y puede o no presentarse en posición de máxima intercuspidación dentaria ^5^.

Se considera oclusión ideal cuando la máxima intercuspidación coincide con la oclusión céntrica, lo que proporciona una mejor masticación. Una oclusión estable depende de la interacción de las diferentes fuerzas que actúan sobre los dientes, por lo que una alteración desfavorable de la anatomía oclusal puede provocar desequilibrio y oclusión traumática. Los cambios biomecánicos inducen a respuestas biológicas de los diferentes componentes del sistema, por lo que las restauraciones dentarias deben ser diseñadas en armonía con los factores guía del sistema masticador, para asegurar que las fuerzas oclusales sean fisiológicamente tolerables para los dientes restantes y las estructuras adyacentes.

La fotoelasticidad es una técnica que permite el estudio de esfuerzos en un cuerpo que se somete a cargas. Es necesario que el modelo tenga propiedades de birrefringencia para que, al observarlo a través de luz polarizada, puedan verse las líneas de color llamadas isocromas, las cuales se asocian con valores de esfuerzo. El análisis cualitativo puede determinar la intensidad de los esfuerzos y observar la dirección que pueden tomar ^6^. 

Dicha técnica ha sido usada por ingenieros para comprender cómo se distribuyen los esfuerzos en estructuras bajo estrés y así predecir posibles rupturas o zonas vulnerables. Este conocimiento es mostrado desde su formación inicial con buenos resultados. En odontología y medicina ha sido utilizado para investigar y analizar materiales, dispositivos o tratamientos ^7^. 

El objetivo del presente estudio es mostrar a través de la fotoelasticidad como se generan esfuerzos durante la preparación dental, así como la forma en que se distribuyen al colocar la restauración. 

## MATERIALES Y MÉTODOS

### Reproducción de dientes

Por medio de moldes de caucho de silicón (P-85Posil, Poliformas de México), se reprodujeron dientes con raíz prefabricados y dientes de un tipodonto tipo Columbia. Después de 36 horas, se retiraron de los moldes. Se cubrieron por dentro con agente desmoldante y fueron vaciados en resina fotoelástica epóxica cristal AWS520, de Epolyglas México, y después de 24 horas se retiraron del molde.

### Obtención de modelos

Se generaron dos tipos de modelos. En uno, se sustituyeron los dientes del tipodonto y fueron fijados con silicón transparente, ya que atornillarlos podría generar tensiones. El segundo se modeló a partir de los dientes en oclusión; se conformó con cera una sección de la mandíbula y el maxilar superior. Se tomó registro de mordida con silicón 3M ™ Imprint™ 4 Bite. Una vez encerado, se tomó una impresión de silicón y, posteriormente, se retiró la cera. Después, se colocaron los dientes en su posición a partir de sus huellas y se volvió a vaciar en resina fotoelástica. Estos modelos se fijaron en un articulador semiajustable con ayuda del registro de mordida. 

### Preparación cavitaria 

En ambos modelos se realizó una preparación dental con fresas de alta velocidad de diamante y carburo de tungsteno. En el segundo modelo se colocó una restauración temporal de óxido de zinc y eugenol con endurecedor (IRM, Dentsply Sirona). Se colocó papel de articular y se simularon los movimientos de apertura, cierre y lateralidad para observar los puntos de contacto prematuros en la restauración, los cuales se eliminaron comprobando la oclusión al realizar nuevamente los movimientos. 

### Registro fotográfico 

Todo el procedimiento se documentó con imágenes, apoyados con un polariscopio. Las imágenes fueron obtenidas colocando los modelos entre las películas polarizadas y con ayuda de la fuente de luz del polariscopio y por fuera del mismo, una cámara CANON EOS 1300D (W) SLR digital sensor CMOS,18,0 Mpx y grabación de vídeo Full HD, anillo inversor 58mm Ø para lente 18-55 3.5 F. Se grabó un video del procedimiento para la ejecución de la cavidad en cada modelo. Se tomaron fotografías antes y después de realizar la preparación, así como después de la colocación de la restauración y en cada desgaste de los puntos prematuros de contacto, todas ejerciendo presión al cierre. 

### Análisis estadístico

Se realizó estadística descriptiva (distribución de frecuencias) y se utilizó la prueba de Wilcoxon para determinar si existía diferencia en la presencia e intensidad de las isocromas antes, durante y después de realizar las preparaciones y la restauración temporal. Los análisis se procesaron en el programa IBM SPSS Statistics V. 22.0.

## RESULTADOS

Se obtuvo la imagen de las isocromas generadas en la resina por compresión y se comparó con las escalas encontradas en la literatura como referencia de la intensidad del esfuerzo. La escala obtenida presenta los siguientes valores: el nivel más bajo de estrés corresponde al primer grupo de isocromas, en el que la intensidad de color y ancho de las bandas es mayor. El nivel intermedio corresponde al segundo grupo de isocromas de color menos intenso. Por último, el nivel más alto de esfuerzo se da a partir del tercer grupo de isocromas, en el que los colores que se notan más claramente son rosa y verde, los demás no se distinguen claramente y el ancho de las bandas es menor (Tabla 1).

**Tabla 1:** Escala con respecto a la literatura ^8^^-^^10^

Cebrián-Carretero, 2012			Pereira, 2018		Pellizzer, 2010		Resina AWS 520	
Isocroma	Escala	Isocroma	Escala		Nivel		
	(N)		(N)				
					Bajo		
						
Negro	0	Negro	0				0
Gris	0.28	Gris	0,28				
Blanco	0.45	Blanco	0,45		Medio		
Amarillo claro	0.6	Amarillo claro	0,6				
Naranja	0.8	Naranja	0,79				
		Rojo pálido	0,9				
Rojo-azul (transición)	1	Rojo-azul (transición)	1				1
Azul oscuro	1.08	Azul oscuro	1,06				
		Azul-verde	1,2				
Verde-amarillo	1.22	Verde-amarillo	1,38				
Naranja	1.39	Naranja	1,62		Alto		
Rosa-rojo	1.63	Rosa-rojo	1,81				
Rosa (transición)	2	Rojo-verde (transición)	2				2
Verde	2.4	Verde	2,33				
Verde-amarillo	2.7	Verde-amarillo	2,5				
		Rojo	2,67				
Amarillo (transición)	3	Rojo-verde (transición)	3				3
		Verde	3,1				
		Rosa	3,6				
	4	Rosa-verde (transición)	4				

### Distribución de esfuerzos durante la preparación dental

**Figura 1:**
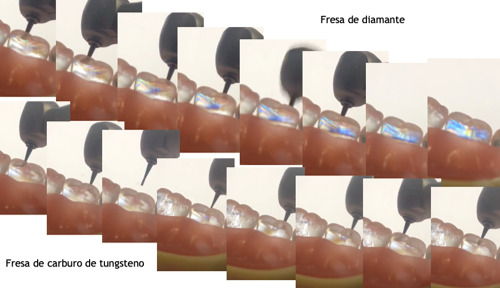
Esfuerzos presentes en los dientes por el trabajo de las fresas de alta velocidad (Autor: Marín, M.)

Al utilizar fresas de diamante, a pesar de ser nuevas, se presentan líneas de concentración que son más intensas que al usar fresas de carburo. Según nuestra escala, al distinguirse el color azul-rosa-amarillo, se asume una intensidad de nivel intermedio. Por otro lado, al utilizar fresas de carburo, solo en dos puntos lograron verse líneas muy tenues en color negro-gris-azul, que son esfuerzos de nivel bajo (Fig. 1), lo que se podría atribuir a la conformación de las hojas de corte. 

Durante el acto clínico, la irrigación evita que el tejido dentinal reblandecido obstruya la zona de corte. En nuestro modelo no se utilizó ningún tipo de irrigación, pero el residuo es polvo, el aire de la pieza fue suficiente para que no se obstruyera, así el corte no generó tanto esfuerzo como en la fresa de diamante. La irrigación cobra mucha importancia para evitar que, además de los esfuerzos generados, se genere calor que pueda dañar la pulpa. 

Este hallazgo es importante y puede requerir mayor profundidad de investigación para determinar mejores técnicas o herramientas para el desbaste de dientes, a fin de minimizar los esfuerzos residuales y, por tanto, el daño a las piezas dentales y el dolor para el paciente.

### Distribución de esfuerzos durante la restauración dental

**Figura 2:**
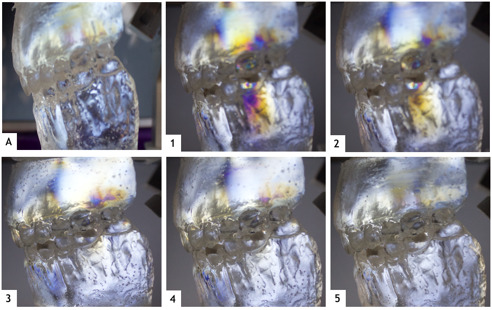
A) Distribución de esfuerzos en el hueso periodontal antes de realizar la preparación. 1 y 2) Esfuerzos en máxima intercuspidación después de obturar con puntos prematuros de contacto presentes. 3, 4 y 5) Progresión del desgaste de los puntos de contacto en la restauración (Autor: Marín, M.)

De inicio, se presentan líneas de concentración en máxima intercuspidación de nivel bajo-intermedio (blanco-amarillo claro) distribuidas en el maxilar superior (Fig. 2a). Durante la preparación cavitaria, en el primer molar inferior, se observó la formación de isocromas en las paredes cavitarias en máxima intercuspidación, con un aumento de intensidad al realizar los deslizamientos laterales y la retrusión. Los esfuerzos no solo son reflejados en la mandíbula; de hecho, son reflejados con mayor intensidad en el maxilar superior, sobre todo cuando la carga de cierre aumenta. Solo al realizar deslizamientos laterales, puede verse esfuerzo en la zona distal del soporte óseo del primer molar en que se realizó la cavidad. 

Ya endurecida la obturación sin una anatomía correcta, se realizaron los movimientos donde el modelo mostró el más alto nivel de esfuerzo (Figs. 2.1 y 2.2). En la zona de la corona, se muestra en nivel intermedio-alto, lo que también se observa en el molar antagonista. A nivel de hueso periodontal debajo de la preparación, se presentan líneas de nivel intermedio, al igual que en la zona distal del hueso periodontal del molar antagonista. Los esfuerzos también son reflejados en zonas más lejanas: entre el canino y el primer premolar inferior de nivel bajo, y entre premolares superiores intermedios. 

Al realizar el desgaste de los puntos de contacto marcados, comienza a verse una disminución de la intensidad de las isocromas hasta lograr una imagen similar a la obtenida antes de realizar la cavidad en máxima intercuspidación (p > 0,05) (Figs. 2.3, 2.4 y 2.5). 

## DISCUSIÓN

Durante la formación profesional que requiere de la observación y el análisis de la fisiología humana, no todos los fenómenos pueden ser explicados en modelos vivos y los anatómicos o virtuales no cubren todas las necesidades. En odontología, uno de los aspectos más difíciles de entender es la biomecánica y la distribución de las cargas en el sistema estomatognático. Es muy importante comprenderlo, ya que la mayoría de los tratamientos odontológicos modifican las condiciones en las que se da esta distribución. Dada la magnitud de las fuerzas que se aplican durante el proceso de masticación y el efecto en el mediano y largo plazo de una alteración en la geometría o dimensiones de las piezas dentales, es crítico tener una herramienta que permita este entendimiento.

En la clínica, la única forma de percibir que existe un problema es por los síntomas que refiere el paciente, al diagnosticar patologías de ATM, trauma oclusal, entre otras, las que en algunos casos pueden detectarse en etapas avanzadas ^(11, 12)^. El origen de algunos de estos problemas podría haber sido el trabajo odontológico, si no se considera la oclusión o la importancia de neutralizar los puntos de contacto prematuro; sin embargo, podría atribuirse el restar importancia a este aspecto al hecho de que, durante nuestra formación como odontólogos, no se tuviera modelos que establezcan una relación directa con este tipo de trastornos. Por tanto, resulta útil generar y usar modelos para hacer evidentes los efectos que tienen las variaciones oclusales provocadas por los tratamientos odontológicos y establecer la relación con la probabilidad de presentar algún tipo de lesión ^13^. El uso de la simulación es una herramienta que potencia el aprendizaje. Según Jaramillo-Naranjo ^14^, mejora el pensamiento abstracto y permite extrapolar el conocimiento, sin experimentar en el entorno real, lo que en nuestra práctica puede evitar acciones experimentales con los pacientes, así como situaciones que pueden ser adversas y con implicaciones éticas. 

Los modelos que se han utilizado para la investigación son muy diversos, algunos enfocados en los dientes, otros ampliados a las arcadas y algunos más complejos incluyen estructuras de cráneo, mandíbula o ligamento periodontal. Todos ellos tienen objetivos muy concretos y han aportado conocimientos valiosos.

Algunos autores han propuesto formas de análisis en modelos fotoelásticos que podrían funcionar bien para resolver la forma de visualizar los esfuerzos generados por diferentes tipos de preparaciones como Butzke ^15^, quien propone un modelo bidimensional de premolares con diferentes tipos de cavidades. Resulta muy ilustrativo ver el cambio en la distribución de los esfuerzos conforme existen diferentes tipos de cavidades. Pereira ^9^ propone un modelo dental para analizar propiedades como contracción y grado de conversión de las resinas compuestas, lo que podría mejorar la comprensión de las diferentes técnicas de colocación y manejo clínico. Otros autores, como Cebrián Carretero ^16^ y Pigozzo ^17^, han trabajado en modelos de cráneo completo con dientes y mandíbula de diferentes materiales, en combinación con los fotoelásticos. Todas estas son propuestas para observar la distribución de esfuerzos de forma más amplia.

Asimismo, Pereira, Cebrián-Carretero y Pellizzer, de quienes tomamos la referencia para la escala, utilizaron modelos fotoelásticos para investigar y analizar situaciones específicas en odontología, como estos u otros parecidos a nuestro modelo, los cuales pueden ayudar a la comprensión en un nivel más básico. Su objetivo es la apropiación de conocimientos abstractos y complejos para hacerlos más sencillos solo por el hecho de percibirlos de forma visual. Nuestro modelo confirma lo que en teoría debíamos saber, pero seguramente en la perspectiva de muchos odontólogos se suele pasar por alto, sobre todo aquellos que recién comienzan y no tienen la experiencia de haber visto las graves consecuencias de no eliminar cuidadosamente los puntos de contacto prematuros, lo que en nuestro modelo es evidente. 

La relevancia clínica radica en comprender que el efecto de las tensiones no fisiológicas originadas por puntos prematuros de contacto pueden estar presentes por causas diversas, incluidas las iatrogenias, y estas pueden asociarse con la presentación de trastornos temporomandibulares, así como lesiones de abfracción y alteraciones pulpares ^18^. 

Existen otros modelos que nos ayudan a observar el fenómeno de distribución de esfuerzos en odontología como el elemento finito ^(19, 20)^; sin embargo, son métodos más difíciles que requieren conocimientos y tecnología especializados, además de que, por las geometrías complejas presentadas en la anatomía humana, exhiben imprecisiones. Aunque puede mostrar ventajas para obtener datos cuantitativos, si se trata de elegir una técnica sencilla y que brinde la posibilidad de mejorar la comprensión a nivel un tanto más cualitativo, la opción recomendada es la fotoelasticidad.

## CONCLUSIONES

Dada la posibilidad de manipular y observar las tonalidades que se generan por las líneas de esfuerzo, el modelo permite comprender cómo las preparaciones y restauraciones deficientes producen reacciones en las estructuras adyacentes, lo que, en el corto plazo, se traducen en dolor para el paciente y, en el mediano plazo, puede generan situaciones más graves.

El modelo permite observar los esfuerzos generados por los procedimientos tanto en los dientes como en el soporte óseo. No es suficiente un solo desgaste para eliminar el esfuerzo excedente y la irrigación también es crucial para reducir el esfuerzo.

Finalmente, dado que este modelo permite tener una visión del dolor que se genera en el paciente, se puede extender su uso a otras aplicaciones, como mejorar procedimientos clínicos y el diseño o rediseño de los instrumentos utilizados. 

## Financiamiento:

Programa de Apoyo a Proyectos para Innovar y Mejorar la Educación de la Universidad Nacional Autónoma de México (PAPIME-UNAM), PE206820
